# The Use of a Smartphone Application in Monitoring HRV during an Altitude Training Camp in Professional Female Cyclists: A Preliminary Study

**DOI:** 10.3390/s21165497

**Published:** 2021-08-15

**Authors:** Alejandro Javaloyes, Manuel Mateo-March, Agustín Manresa-Rocamora, Santiago Sanz-Quinto, Manuel Moya-Ramón

**Affiliations:** 1 Sports Research Centre, Department of Sports Science, Universidad Miguel Hernández de Elche, 03202 Elche, Spain; ajavaloyes@umh.es (A.J.); amanresa@umh.es (A.M.-R.); atleta@santiago-sanz.com (S.S.-Q.); 2 Spanish Cycling Federation, 28008 Madrid, Spain; manuel.mateo@ymail.com

**Keywords:** endurance training, monitoring, elite sport, professional cycling

## Abstract

Altitude training is a common strategy to improve performance in endurance athletes. In this context, the monitoring of training and the athletes’ response is essential to ensure positive adaptations. Heart rate variability (HRV) has been proposed as a tool to evaluate stress and the response to training. In this regard, many smartphone applications have emerged allowing a wide access to recording HRV easily. The purpose of this study was to describe the changes of HRV using a validated smartphone application before (Pre-TC), during (TC), and after (Post-TC) an altitude training camp in female professional cyclists. Training load (TL) and vagal markers of heart rate variability (LnRMSSD, LnRMSSDcv) of seven professional female cyclists before, during, and after and altitude training camp were monitored. Training volume (SMD = 0.80), LnRMSSD (SMD = 1.06), and LnRMSSDcv (SMD = −0.98) showed moderate changes from Pre-TC to TC. Training volume (SMD = 0.74), TL (SMD = 0.75), LnRMSSD (SMD = −1.11) and LnRMSSDcv (SMD = 0.83) showed moderate changes from TC to Post-TC. Individual analysis showed that heart rate variability responded differently among subjects. The use of a smartphone application to measure HRV is a useful tool to evaluate the individual response to training in female cyclists.

## 1. Introduction

In high-level endurance athletes, altitude training camps are a common strategy to improve performance [1]. The main utility of these training camps resides in the signaling of hypoxia-inducible factor-1α, which stimulates the erythropoietic response and consequently, favors the hemoglobin mass and other hematological and non-hematological changes [2,3,4]. These changes are related to the performance in aerobic disciplines because of the use of oxygen as the main source of energy. According to Mujika et al. [5], the proper programming of altitude training camps is the key factor to successfully obtain the desirable adaptations to achieve performance increases. In general, it seems that a duration of 3 to 4 weeks at 2000–2500 m of altitude is optimal [6]. In altitude training camps, training periodization must be prescribed to maximize and favor the benefits of the hypoxic condition. Therefore, training volume, intensity, and daily and weekly training frequency should be properly adjusted to match the additional stress of altitude [5]. Thus, the selection of proper tools to assess the stress-recovery process could be useful to optimize the training process, maximize the benefits of altitude training camps, and prevent non-functional overreaching [7].

In this regard, Heart Rate Variability (HRV) has been suggested as a tool to assess fatigue, stress, and response to the training load (TL) [8]. HRV reflects the autonomous nervous system (ANS) status and reflects the adaptive response through the sympathovagal balance [9]. Many studies have used HRV to monitor the adaptation to training at sea levels and they conclude on its usefulness [10,11,12]. It has been reported that HRV decreases after an overload training period, showing that the additional training stress results in a decrease in parasympathetic activity [13]. Likewise, it seems that after a recovery or a decrease in training, there is an increase in HRV [13]. Some studies have linked an increase in HRV with a positive response to training and an increase in performance [14]. Furthermore, significant and prolonged decreases in HRV are related to a poor or bad response to training and non-functional overreaching [15]. However, some authors did not find direct relationships between TL and HRV [16,17,18]. Barrero et al. [17] did not report a correlation between an increase in the TL and HRV in well-trained female cyclists that performed the Tour of France circuit. In the same line, another research did not find a relationship between readiness-to-perform and HRV [12], concluding that HRV presents a highly individual behavior and its interpretation must be individualized. This fact could be due to the fact that HRV is influenced by all kinds of stress, not only by the training stress. The hypoxic condition in altitude is stressful for humans and the body’s ability to overcome this stress produces some adaptations, like the aforementioned adaptation. In this regard, a decrease in HRV in hypoxia conditions has been reported [19,20,21]. Thus, the additional stress imposed by altitude may be harmful during training camps if the TL and the body’s response to the TL and altitude are not properly monitored. Schmitt et al. [22] proposed the daily adjustment of TL monitoring based on HRV morning recordings during an altitude training camp in elite endurance athletes. They found that adjusting the TL based on HRV will blunt the decrease in parasympathetic activity compared with a predefined training program. This confirmed that the use of HRV may be helpful in the prevention of overtraining syndrome, even in altitude conditions.

In the last years, the measurement of HRV has been simplified thanks to technological and scientific developments. For example, measurement length has been shortened from short (5 to 10 min) to ultra-short (<1.5 min) [23,24]. Furthermore, many smartphone applications have emerged, allowing a wide access to recording HRV easily [25]. In this regard, HRV4training is a scientifically validated smartphone application [26] that can register the HRV from a normal Bluetooth chest strap or use photoplethysmography (PPG) technology. PPG measures heart rate through the reflection of the illumination of the skin when a light-emitting diode is applied (e.g., the smartphone’s camera flash), as local blood volume increases during cardiac systole, which reduces the light intensity and therefore detects the heartbeat. The PPG measures provided by the HRV4training smartphone application have shown nearly perfect correlations against the gold standard electrocardiogram [26]. Thus, PPG simplifies the daily monitoring of valid HRV measurements, as no additional devices are required like heart rate chest straps or electrodes. This fact could lead to better compliance with the daily measurement of HRV.

Although HRV4training has been implemented in different studies aiming at the optimization of training monitoring [10,27,28], to the best of our knowledge, there is no study in female professional cyclists reporting the response of HRV using HRV4training during an altitude training camp. Therefore, the purpose of this study was to describe the dynamic of HRV parasympathetic markers before, during and after an altitude training camp of female professional cyclists using the PPG technology of this smartphone application.

## 2. Materials and Methods

### 2.1. Subjects

Seven (*n* = 7) female professional cyclists participated in this study. All cyclists were fully informed about the purpose of the study and remained healthy and free of injury during the duration of the study. All the subjects belonged to the same professional cycling team. The research followed the principles of the declaration of Helsinki and was approved by the ethical committee of the Miguel Hernandez University of Elche (DPS.JSM.02.18).

### 2.2. Design

An observational study design was carried out during nine weeks subdivided into three periods of three weeks: (1) Pre-altitude training camp (Pre-TC), (2) Altitude training camp (TC), and (3) Post-altitude training camp (Post-TC). The cyclists maintained their individualized training program during the nine weeks. The TL followed the previous recommendations [5] to ensure the best possible acclimatization to high-altitude conditions. During Pre-TC and Post-TC, the training altitude and residence of the cyclists were <1000 m from sea level. There were brief exposures at higher altitudes due to the nature of road cycling (i.e., mountain climbs during training). During the TC, cyclists were living at an altitude between 2000 and 2300 m from sea level and spent most of the day (>18 h) at this altitude. The inclusion criteria were that all the participants should be female cyclists currently racing within a professional cycling team. To be included in the final analysis, the cyclists must complete at least the 90% of the HRV measurements with no less than 6 measurements each week.

### 2.3. Methods

The TL was continuously monitored with a Garmin 820 (Garmin Ltd., Southampton, UK) that recorded training volume and intensity. Training intensity was monitored using power output measured with a portable power meter fitted on the crankset (Power2Max type S, Zossen, Germany) [29] and training load was calculated with training stress score (TSS) mathematical model [30]. This model calculates training load using the time (volume) and intensity (power output) by the following equation:

TSS (arbitrary units) = ((t⋅NP⋅IF)/(FTP⋅3600)) ⋅100(1)

where t is the training volume in seconds, NP is the normalized power attained during the training, and IF (intensity factor) is the ratio between the NP and the functional threshold power (FTP, i.e., 95% of the highest 20-min mean power output obtained during the training sessions, tests, and competitions of the preceding four weeks) [31]. The TSS metric was expressed in arbitrary units (a.u). All the training data was analyzed daily using specific training software that included a cloud service (TrainingPeaks, Bolder, EEUU).

For the daily HRV assessment, the participants were instructed to measure their R-R interval data upon waking up and emptying their bladder. The HRV measurements were captured with the HRV4training smartphone application [26]. HRV was measured in a supine position and over a 90 s period [24]. Cyclists were instructed to remain seated and not to perform any further activity during the recordings. The first 30 s were discarded and the last 60 s of the HRV measurement were captured [32]. The root mean squared differences of successive RR intervals (RMSSD) was chosen as the vagal index, based on its greater suitability and reliability than other indexes [33]. The HRV data were transformed by taking the natural logarithm (LnRMSSD) to allow parametric statistical comparisons that assume a normal distribution [34]. In addition, the coefficient of variation of LnRMSSD (LnRMSSDcv) was calculated because of the relationship of this variable with a positive or negative response to training. When HRV data were inappropriate or erroneous, the cyclists would be informed by the smartphone application and the data would be discarded. Then, the cyclists would be asked to repeat the measurement until it was deemed successful.

### 2.4. Statistical Analysis

A Shapiro–Wilk test was used for testing the normality of the data. Besides, the normal distribution of the data was graphically verified by box plot and Q-Q graphs. All variables were reported as mean ± standard deviation (SD). A repeated-measures analysis of variance (ANOVA) was performed to analyze the effects of the training period on dependent variables. Mauchly’s sphericity test was used to validate repeated-measures ANOVA, and the Greenhouse–Geisser correction of the F statistical degree of freedoms was implemented in case of no assumption of sphericity. Later, post hoc comparisons were carried out using pairwise comparisons with a Sidak correction for multiple comparisons. The critical value used to consider statistical significance was set at *p* < 0.05. Standardized mean difference (SMD) was used to estimate the magnitude of changes between training periods. SMD was interpreted as follows: trivial (<0.20), small (0.20–0.59), moderate (0.60–1.19), large (1.20–1.99), and very large (>2.0). Pearson’s correlation was used to quantify the relationship between training load and autonomic variables. Pearson’s correlation was interpreted as follows: weak (<0.30), moderate (0.30–0.60), and strong (>0.60). All data analyses were carried out using IBM SPSS Statistics software (v.25; IBM Corp., Armonk, NY, USA). The individual changes were assessed by computed Pre-TC as the baseline period and calculating the percentage of change from Pre-TC to TC and Post-TC.

## 3. Results

Training volume, intensity of training, and TL did not change (*p* ≥ 0.05) from Pre-TC to TC (Table 1). Training volume showed moderate increases during the TC (SMD = 0.80) while total distance covered showed moderate decreases (SMD = −1.04). In contrast, distance (*p* = 0.01; SMD = 2.842) increased from TC to Post-TC. Training volume (SMD = 0.74) and TL (SMD = 0.75) showed moderate increases from TC to Post-TC.

LnRMSSD did not change statistically from Pre-TC to TC, but it showed moderate increases (SMD = 1.06). Conversely, LnRMSSDcv showed moderate decreases (SMD = −0.98) in the same period. From TC to Post-TC, LnRMSSDcv increased (*p* = 0.02; SMD = 0.83). LnRMSSD also showed moderate decreases (SMD = −1.11). 

**Figure 1:**
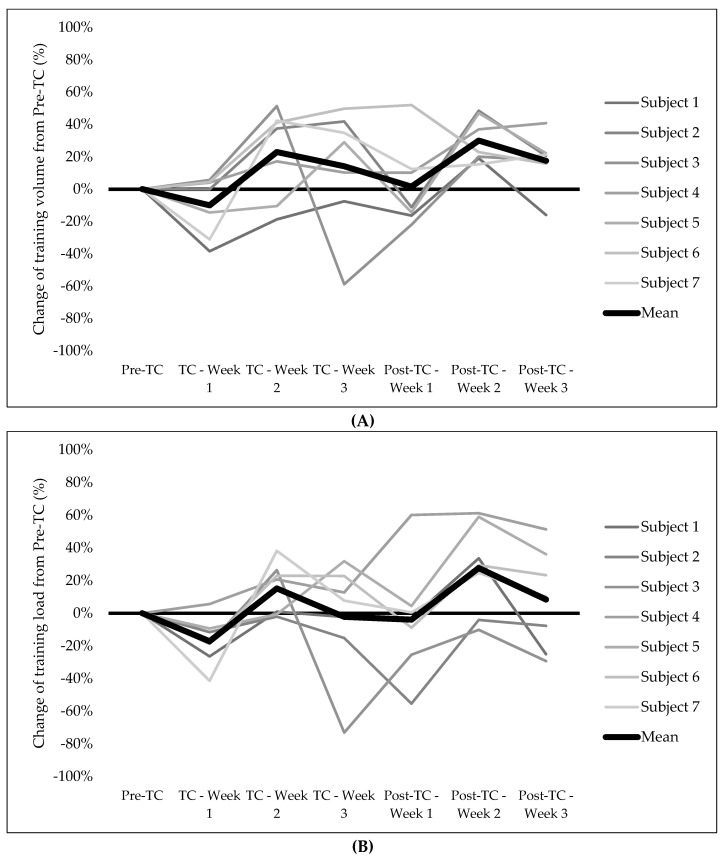

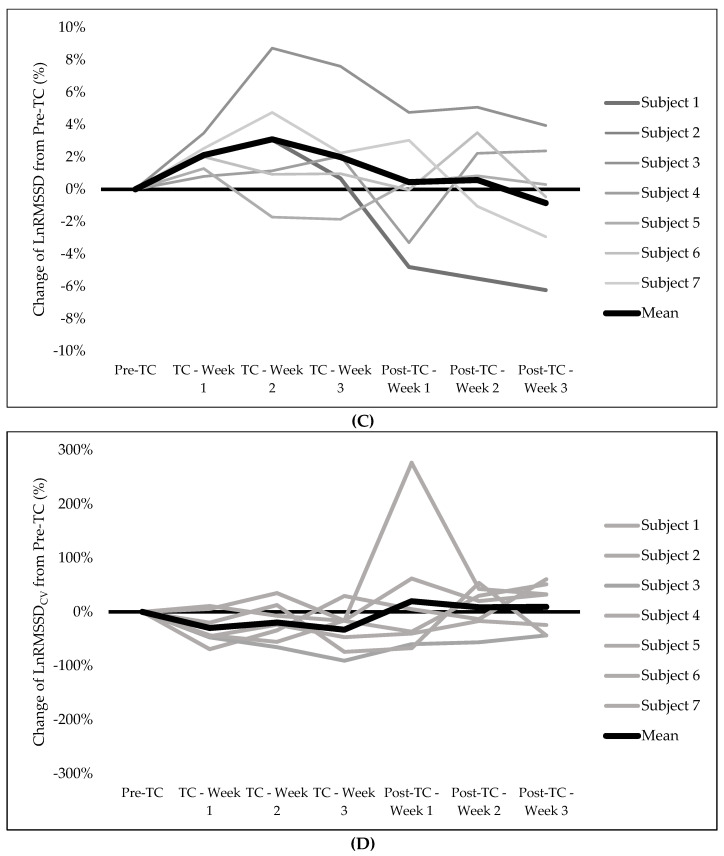
Individual changes (%) from Pre-TC to TC and Post-TC in training volume (**A**), training load (**B**), LnRMSSD (**C**) and LnRMSSDcv (**D**). Pre-TC: Period of 3 weeks before the altitude training camp; TC: Period of 3 weeks of altitude training camp; Post-TC: Period of 3 weeks after the altitude training camp; LnRMSSD: Natural logarithm of the root mean squared differences of successive RR intervals; LnRMSSDcv: Coefficient of variation of the natural logarithm of the root mean squared differences of successive RR intervals.

Figure 1 shows the individual changes (in percentage) during the TC and the Post-TC in training volume (A), training load (B), LnRMSSD (C), and LnRMSSDcv (D). The training volume (Figure 1A) changed by −10% in the first week of the TC. These changes ranged between 4% and −38% among the subjects. The TL (Figure 1B) was changed during the first week of TC by −17%, this variation ranged between 6% and −41% among subjects. After the first week of the TC, training volume and TL tend to increase during the remaining weeks of the TC and the Post-TC. However, high differences were found among subjects. LnRMSSDcv showed a mean change of −30% during the first week of TC and the individual change ranged between 6% and −69%.

LnRMSSD showed weak and non-significant (*p* > 0.05) correlations with training volume (r = 0.18), distance (r = 0.11), and TL (r = 0.15). Similar results were observed with LnRMSSDcv that did not correlate with training volume (r = 0.01), distance (r = 0.13), and TL (r = 0.05).

## 4. Discussion

This study aimed to describe the influence of training load and altitude in the HRV response of professional female cyclists using the HRV4training smartphone application. The main finding of this study is that HRV responded differently among the cyclists and the use of HRV4training may help coaches and practitioners to individualize training load and recovery to optimize training adaptation. 

HRV is a non-invasive methodology used to assess the status of the autonomous nervous system [35]. In this regard, a significant increase in the stress imposed on the athlete will produce a decrease in the parasympathetic activity [36]. Conversely, the recovery processes and positive training adaptation will increase parasympathetic activity. In our study, moderate increases in training volume (SMD = 0.744) and TL (SMD = 0.754) during the Post-TC resulted in moderate decreases in LnRMSSD (SMD = −1.111) with concomitant increases in LnRMSSDcv (SMD = 0.827). These results can be observed in detail in Table 1. These findings are in line with previously published articles that showed a reduction of LnRMSSD and increases in LnRMSSDcv during overload periods in collegiate female soccer players [27] and international female hockey players [37]. Thus, it seems that these vagal markers could be used to track the response to the training of professional female cyclists both at sea level and in moderate altitude conditions using a smartphone application. The implementation of daily HRV measurements in the field is possible because of the use of validated smartphone applications. In this regard, the HRV4training smartphone application has shown nearly perfect correlations with laboratory ECG measurements [26]. The HRV4training smartphone application allowed coaches to measure HRV accurately in ecological sport contexts.

In contrast, moderate increases in training volume from the Pre-TC to the TC result in moderate increases of LnRMSSD and moderate decreases of LnRMSSDcv, despite the altitude level during the TC (>2000 m above sea level). A possible explanation for this fact is that the TL remained constant between the Pre-TC and the TC (see Table 1) and the moderate levels of hypoxia did not produce enough stress to affect their vagal tone. To note is that this population had previous experience in altitude training camps and previous research has shown that this experience may produce lesser disturbances in the athletes’ body. Furthermore, this population usually performed a large volume of training with high proportions of high intensity training [38]. This fact may cause that the response to the additional stress imposed by altitude is not enough to produce changes in HRV when the TL remained similar (no increases and decreases in the TL) as in this study, especially in moderate altitudes in which the training camps for endurance athletes took place (1800–2500 m). Therefore, in this study vagal markers were affected when the TL changed, but not with changes in altitude or training volume. This fact is important because the TL is the combination of training volume and intensity of effort. Thus, in the case of changes from the Pre-TC to the TC, total TL remained similar despite a decrease in training volume.

In this observational study, the subjects followed their individualized training program. As can be seen in Figure 1A, the training volume changed by −10% in the first week of the TC. However, this variation from the Pre-TC to the first week of the TC ranged between 4% and −38% among the subjects. Regarding the TL (Figure 1B), the first week of the TC changed by −17%; this variation ranged between 6% and −41% among the subjects. Mujika et al. [5] recommended reductions of the training volume and load during the initial days of an altitude training camp. However, this recommendation may vary among different subjects depending on different factors such as their previous experience in altitude training or their individual response to hypoxia. During the following weeks of the TC and the Post-TC, the training volume and the TL tended to increase but with high differences among all the subjects. The highest increases in training occurred during the last weeks of the Post-TC.

LnRMSSD increased during the first week of the TC in all the subjects despite the altitude (Figure 1C). The mean increase was a 2% while the individual change ranged between 1% and 3%. This could be due to the reduction of the training volume and the TL. Similar research but at sea level has shown greater increases in vagal markers of HRV when the TL is reduced after an overload period [13]. In this study, it is possible that the moderate altitude resulted in a lower increase in LnRMSSD; however, this is speculative, and this result must be taken with caution. 

LnRMSSDcv showed a mean change of −30% during the first week of the TC and the individual change ranged between 6% and −69%. Thus, it seems that the exposition to moderate levels of hypoxia did not produce enough stress to induce reductions in the vagal activity in this population and this reduction could be due to the concomitant reduction in the TL, as seen before in other sports [37,39,40]. It is important to note that these markers evolved differently among individuals. For example, subject 5 experienced a reduction of LnRMSSD during weeks 2 and 3 of the TC. In contrast, subject 3 showed the inverse response with large increases (8–9%) during the same mentioned weeks (see Figure 1C). Therefore, the use of these vagal markers could be used to detect the different responses during the training and, consequently, individualize the training prescription to optimize increases in performance [41] and avoid overtraining [42].

Regarding correlational analysis, our results support previous studies that did not find relationships between HRV parasympathetic markers (LnRMSSD and LnRMSSDcv) and training load variables [16,17,18]. These results reinforce the hypothesis about the individual response of HRV for each athlete and the need for individual analysis and interpretation of the daily HRV measurements.

The nature of this preliminary study was observational, in which researchers described the changes of HRV due to the changes in the TL and/or altitude in female professional cyclists in an ecological context. Thus, this study has limitations that must be acknowledged. The main weakness of this study is the low sample size (*n* = 7); for this reason, the authors also showed the individual change of each subject in the variables analyzed in this study. Furthermore, the subjects followed their individualized training program with their own overload and recovery periods. Further studies should standardize the training load among subjects to discern clear patterns in this population. However, as cyclists followed the scientific evidence to optimize altitude training camps, the TL dynamics were very similar among them. In addition, future studies should add direct performance measurements to further support the relationship between the HRV dynamics and the changes in performance. In addition to this, blood hematological response should be taken into account to evaluate the effectiveness of the response to altitude training. It is worth mentioning that all participants are from the same team, so the results may differ from the global reality of other athletes. Given the limitations of this preliminary study, the results and conclusions must be interpreted with caution. Our future work will continue investigating the use of HRV in this population but solving these limitations.

## 5. Conclusions

This study concludes that the use of a smartphone application to measure HRV (HRV4training) could be a useful tool to evaluate the individual response to training in professional female cyclists. The information given by HRV4training can be used by coaches and practitioners to optimize the training process in this elite athlete population.

## Figures and Tables

**Table 1:** Cardiac autonomic measures, training load values and statistical comparisons.

	Pre-TC	TC	Post-TC	Statistical Comparisons								
							Pre-TC vs. TC		TC vs. Post-TC		Pre-TC vs. Post-TC	
				F	*p*	*η^2^*	*p*	SMD	*p*	SMD	*p*	SMD
Training volume (min)	16,1245.00 ± 7989.85	174,100.00 ± 10008.96	185,993.00 ± 6449.65	4.195	0.042	0.411	0.656	0.804	0.284	0.744	0.041	1.548
Distance (km)	1209.90 ± 77.30	1095.70 ± 64.10	1407.31 ± 42.99	14.474	<0.001	0.707	0.343	−1.042	0.005	2.842	0.024	1.801
Intensity (%)	76.95 ± 1.44	76.09 ± 1.73	77.12 ± 3.10	0.096	0.910	0.016	0.979	−0.182	0.962	0.218	0.999	0.035
TL (au)	2926.48 ± 183.19	2969.94 ± 100.18	3286.35 ± 184.83	1.536	0.255	0.204	0.993	0.104	0.379	0.754	0.605	0.858
LnRMSSD	4.67 ± 0.08	4.76 ± 0.07	4.66 ± 0.11	2.756	0.104	0.315	0.179	1.063	0.229	−1.111	0.999	−0.045
LnRMSSDcv	5.65 ± 1.26	3.64 ± 0.49	5.33 ± 0.61	1.947	0.185	0.245	0.367	−0.980	0.024	0.827	0.995	−0.153

Values are delivered as mean ± standard deviation. Statistical significance was set at *p* < 0.05. F = F value. *η****^2^*** = eta squared. TL: training load; LnRMSSD: Natural algorithm of root mean squared differences of successive RR intervals; LnRMSSDcv: Coefficient of variation of the natural algorithm of root mean squared differences of successive RR intervals.

## Data Availability

The datasets generated from the current study are available from the corresponding author on reasonable request.
